# Surgical Diseases of Women—Granulation and Ulceration of Cervix Uteri

**Published:** 1864-07

**Authors:** J. F. Miner


BUFFALO

### Medical and Surgical Journal.

VOL. III. JULY, 1864. No. 12.

ART. I.—*Surgical Diseases of Women—Granulation and Ulceration of Cervix Uteri*—By J. F. Miner, M. D.

Surgical Diseases of Women, so-called, include a long list of the more common and more important diseases of females; diseases which are mainly local in character, and which require for treatment local applications rather than general medication. It will be the object to consider briefly some of these diseases, their causes, pathological conditions and the means which we possess for their relief or cure. This will be done with the view of separating the valuable from the worthless, and reducing the mass of mixed truth and error which has accumulated to more consistent and available proportions. Inquiries from correspondents, constantly impress the belief that though much has been written upon this subject, still practitioners are not able readily to determine what may, and what may not, be done for the relief of many of the conditions included under this head; there is perhaps more difficulty in ascertaining what may not properly be attempted, than what may, and a constant distrust is manifested, induced mainly by the feeling that some plan of cure, or article of application or instrument for appliance, is successful in other hands, which in their own is injurious.

There are great differences of opinion among medical men upon the subject of uterine diseases, one class holding that the actual lesions of this organ have not an independent origin, but are the result of diseases of other organs, or of the general system, and that the accompanying symptoms usually present in actual disease of the uterus do not depend upon this disease at all, and may be removed by attention to the general system or without any treatment especially directed to the condition of the uterus.

On the other hand it is held, that the diseases of the uterus exercise immense influence over the whole system through the spinal nerves; and that the only rational and successful method of cure, is found in measures adapted to rectify the diseased condition of this organ. This latter class either hold that the sympathetic influence of this organ is only manifested when inflamed or ulcerated, or maintain that the inflammation and ulceration are of slight, if of any importance, and that the whole difficulty arises from some sort of displacement. Inflammation, ulceration, metritis, endo metritis and the various *versions* constitute the pathological conditions upon which hang the great burden of female woes, according to the views of both parties who believe in independent local change. It is not with the view of taking sides in these controversies, that the opinions of authors have been mentioned, but with the hope of remaining impartial and of presenting the results of experience and observation unbiased by prejudice. It may be well however to say in passing, that unquestionably neither of the parties referred to are wholly correct, the truth laying between, rather than at either extreme, and extending somewhat on both sides. It can hardly be doubted that general conditions of the system do sometimes predispose to uterine irritation and inflammation, certainly it will not be denied that uterine diseases disappear under favorable general influences and without any local medication. That the subsidence of local disease is due to improvement in general health and vigor often appears probable, and this has led some to suppose that the local lesion was the effect of general disease, springing from it as a cause and depending upon its removal as a cure. To commence the treatment of manifest local change by the administration of medicine, which, to say the least, is of doubtful influence even over the general system, and to trust exclusively to this, as the main and only available means of cure, is shutting our eyes to palpable truth and combatting disease with uncertain weapons, while the instruments of control are within our grasp. Before estimating however the value of our remedies, either local or general, we should remember that nature, with an Almighty power, is operating to restore health by influences which we do not fully understand, but having definite laws and working successful issues, which we often erroneously ascribe to our remedies.

Congestion, inflammation, ulceration—different stages of the same original disease, with its sub-divisions into endo-metritis, endo-cervicites, complicated by the flexions and *versions*, express the morbid conditions present in almost all cases—in at least the great majority of instances of uterine disease. Inflammation then, in its various stages and locations with its complications, is *the* disease the treatment of which has given rise to a specialty in practice, and been thought difficult of detection and stubborn of cure. Of course various other diseases are also included in the scope of such specialty when presented, but the treatment of this one, constitutes so large a proportion of the cases presented that the others are only exceptional, and come nearly as much within the scope of the general practitioner of medicine and surgery as of the uterine specialist.

All that is known of this disease—its causes, diagnosis and treatment—may be acquired by every physician with the greatest ease. It is not obscure and uncertain in its general symptoms, while its local conditions are plain and obvious; even the inexperienced practitioner may readily distinguish its nature, and properly administer to its cure. It is principally with the view to simplify and render more plain a large class of cases over which hangs, or has been supposed to hang some mystery, that this article is written. These diseases have indeed been obscured by the curtain of false modesty, sometimes, alas! by prejudice and indolence, but aside from this, there is no intrinsic difficulty of diagnosis, and the treatment is more simple and successful than in most of the diseases which depend in any degree upon medication for favorable termination.

It will appear remarkable that so many different conditions should be classed under one general head and considered in any degree as one disease; but a little reflection and experience it is believed will convince of the propriety and consistency of such view. We sometimes meet cases of displacement, *retroversion* for instance, without any accompanying inflammation, but these cases are comparatively rare, and when found are of little importance, since retroversion without inflammation is productive of no inconvenience other than arises from pressure upon the rectum, nerves or blood vessels, and, in some instances this is not cause of complaint. Passive, subacute or chronic inflammation is the almost universal form observed, while acute inflammation is rarely seen, except after labor, abortion, or the sudden arrest of menstruation, and will not be considered at present only in its relation to subsequent chronic disease.

When it was discovered that the uterus sometimes had versions and flexions, it was at once imagined that everything was explained. If Simpson's sound demonstrated version or flexion, diagnosis was complete and wholly satisfactory; patient and doctor rested from further investigation; we wish we might add, from further interference. The os uteri was tender, and irritable, cervical canal open, and granular, and numerous general and local evidences of uterine disease present, but the one word "retroversion" included everything, and at the same time indicated necessity of rectifying the mal-position. Here commenced the pessary age. Now, when its use has nearly passed away, or is much more confined to its legitimate scope, is a poor time to speak of its abuse; it is well however sometimes to confess our errors even if too late to rectify their injuries. The pessary system of practice is not so completely abandoned that estimation of its benefits may not be useful, nor is *retroversion, anteversion*, etc. so well understood that it is not constantly regarded as the primary uncomplicated disease; sufficient in itself to account for every conceivable uterine symptom. It is well understood that great differences of opinion prevail upon these points, with men of equal ability and experience. This will not appear remarkable when we reflect upon the nature of these diseases, and their terminations under different and opposite plans of treatment. We are, no doubt, sometimes misled, by nature accomplishing cures, while yet vigorously opposed by remedies.

Having already trespassed upon the reader by the length of our preliminary remarks, we proceed to give in brief the *symptoms, causes, local changes*, and *treatment for chronic inflammation of the uterus.*

*General Symptoms of Uterine Inflammation.*—These are exceedingly numerous and varied; all diseases of the uterus affect other and distant organs in greater or less degree, and these sympathetic disturbances are greatly similar in character, whatever may be the nature of the uterine affection. Diseases of this organ, the most dissimilar produce the same general symptoms, and consequently diagnosis cannot be made from these indications alone. Most, or all, of the functions of the general system are more or less interfered with; many times the slighter lesions producing the severer constitutional disturbances. The stomach is perhaps more frequently affected than any other organ. We see it in pregnancy and in every form of uterine disease. We have anorexia and voracity, sometimes loathing of food, or great desire for the most disgusting articles; nausea and frequent vomiting; indigestion, and all the numerous forms of discomfort which attend dyspepsia. Bowels are constipated or relaxed; dejections natural and proper, or watery, profuse, deficient, dry or hard. Mucous diarrhœa is often observed, and the bowels are distended by gas, and disturbed by its passage from one part of the bowels to another; distension of the abdomen is often so great as to induce the belief of pregnancy.

Of all the conditions of the bowels not one exerts so prejudicial influence over local disease as large fæcal accumulation, which sometimes seems to be, and perhaps is, the cause of the uterine disease, rather than caused by it. In cases of retroversion, obstruction of the bowels is sometimes caused by the pressure. The bowels are necessarily much influenced by the secretion from the liver, and this organ is thought to be often affected in its action by sympathetic influence and stimulated to profuse secretion of bile or rendered torpid in its action, inducing deficiency. These conditions of the liver, though often present, are not so certainly traced to uterine disorder as most of the disturbances we include under the head of general symptoms of uterine disease.

The *heart* is disturbed in its action nearly as frequently perhaps as any organ, and the advice of the physician is often asked from apprehension of organic disease. Palpitation and pain in the præcordial region are the more common symptoms complained of, though intermission in the heart beat, and great irregularity in the distribution of the blood are often observed; these symptoms are paroxysmal, and generally induced by some unusual excitement. It is quite common to find the feet and hands cold, and see them remain so for hours, while the face is flushed, head hot, and perhaps also other parts of the system; the heat extending down the spine, and being especially complained of in the lumbar region. Many other disturbances in the circulatory system are occasionally observed; the force of the heart is sometimes so lessened, that alarming symptoms are produced by fright, surprise, grief, or other emotions.

The *kidneys* are particularly liable to sympathetic disturbance, and the urine to become profuse and limpid, or scanty and highly charged with the salts, which may be in proper proportions, or one may be in undue quantity in relation to the others. The urine is often secreted in small quantity and its discharge attended by pain. It is often retained and great distension of the bladder produced unless attention is directed to its condition. In some cases extension of inflammation to the bladder and urethra cause great pain in micturition or immediately after, though this is often complained of, when there is no ground to suppose that the pain is from inflammation, but is sympathetic merely,

There is great sensitiveness in many parts; the bowels are tender and sensitive on pressure, the surface generally appears sensitive, particularly is this noticable in the spinal region; pressure upon the vertebræ causes pain in so great degree that physicians have been misled by this symptom alone.

"Spinal irritation" or "spinal disease," has been found in thousands of instances, when a better acquaintance with the symptoms of uterine inflammation or congestion, would have led to a more correct diagnosis. This disease is not as fashionable now as formerly, when to the error of diagnosis was added the barbarity of practice common in the days of religious persecution; they were cupped, leeched, blistered, pustulated; burned, in a word they were "burned, torn by wild beasts, sawn asunder, persecuted, tormented;" they were not cured, though they sometimes recovered from the disease, and more remarkable still, from the effects of treatment.

The *nervous system* suffers most in uterine affections, and through it are produced some of the most severe and troublesome complications. *Spasms and hysterical convulsions* are apt to appear upon unusual excitement; they are distressing symptoms of uterine disorder, are exceedingly varied in form and severity, and show great irritability of the nervous system. These symptoms show themselves in paroxysms, which are often repeated.

The *mental* faculties are greatly affected during the existence of uterine disease; this has been noticed in rare instances ever since uterine disease has been recognized; recently attention is more than ever directed to this point. It is now being claimed by some special workers in uterine pathology that insanity in women is caused by inflammation, irritation, or other uterine disease, in a large majority of cases. There can be no doubt that the sexual and uterine system exert a great influence over the mental operations, while it is equally certain that insanity cannot with our present modes of investigation be traced back to tangible, local, organic change in the uterus or appendages in a majority of cases. Puerperal mania is sometimes complicated with uterine inflammation or irritation, with patulous ulcerated or granular condition of the neck and os uteri; two cases are now under treatment in the Buffalo Asylum with this condition. But mania is often present when no known method of examination can detect any uterine change, and when these conditions are found, it is not easy to estimate the dependence of insanity upon it, as a cause. These conditions may even be present and apparently cured by local applications, the insanity at the same time gradually passing away, and still the evidence is only presumptive that insanity was caused by the uterine condition or cured by its removal. Puerperal mania terminates favorably according to statistics, in great majority of instances, and has done so without our once even looking for any local change in the uterus. This may be accounted for in the great tendency of such acute cases of disease to terminate spontaneously in recovery. There seems to be a wonderful relation between the sexual system and the intellectual; the mind is no doubt greatly influenced in its operations by conditions of this system which we do not understand. Mania is caused, or there is reason to believe it caused, by functional derangements of the uterine system, much more frequently than organic. Cases are not rare, where chlorotic girls have violent mania which subsides and forever disappears upon the appearance of the menstrual secretion and general improvement of the system. Mania is sometimes observed in girls at puberty, and often in women at the cessation of menstruation. Unnatural excitement from masturbation and excessive indulgence in sexual connection or unnatural chastity are common causes of insanity. Insanity is sometimes associated with inflammation, congestion and ulceration of the uterine neck, and it appears probable that such local disease acts in some cases at least as a principal cause of the mania.

There are a great many other sympathetic disturbances of the general system; indeed it is remarkable how numerous and varied are the general symptoms of uterine disorder. They require to be understood, though very little definite knowledge of the specific disease can be learned from them; they simply point to disturbance of the uterine system as their source and cause, but do not indicate the form of disease present.

*Local Symptoms of Uterine Inflammation.*—Pain and heat in the sacral region; tenderness in the lower portion of the abdomen; discomfort in assuming erect position; feeling of weight in the pelvis; leucorrhœa; painful micturition; deranged menstruation; to which may be added the conditions observed in the cervix and os uteri, viz: enlargement, congestion, abrasion of the mucous membrane, ulceration, induration, softening, tenderness, atrophy the result of inflammation, and the various displacements and versions. These conditions are caused by inflammation in the body or neck of the uterus, which may effect the mucous membrane in any portion, or may extend to the sub-mucous tissues.

The more common causes of this inflammatory action is pregnancy, abortions and labor, severe exercise, cold, constipation, suppressed menstruation, sexual indulgence, to which might be added other, and less easily traced, but not less operative causes.

*Means of Diagnosis.*—At the present time there is no excuse for neglecting physical examination in cases of supposed disease of the uterus, by which we may arrive at positive knowledge of the extent and nature of these affections. It is a low and imperfectly educated female, who objects, when suffering from such disease, to a careful and thorough examination, and it is a fastidious, flimsy, unmanly, ignorant and incapable physician who consents to continue the care of females with the general symptoms of uterine disturbance, without instituting such examination. It should be done thoroughly, by good light, in proper position, with suitable instruments; at the same time it should be done with the reserve of a gentleman and the delicacy of a true physician.

By the *touch* we may learn the condition of the rectum, if free from hemorrhoidal tumors or fæcal accumulations; the bladder, if it is inflamed or contains calculous or other foreign substance; the whole pelvis, if it contain tumors or any sort of unusual conditions. Especially we may learn the condition of the uterus, its position, shape of the neck and body, sensitiveness, hardness, patulence of the os, and if from the os or cervix are protruding any unnatural growths; the weight of the organ may be also estimated.

With the *speculum*, (which should always be used of as large size as the case will admit,) we shall be able to see many conditions of disease, and to make direct application to surfaces which are thus fully exposed. We shall see irritable, bleeding, granular, ulcerated surfaces, patulous, indurated, or otherwise diseased conditions, which depend upon congestion and inflammation as a cause. The most common lesion, that is the stage of inflammation in which advice of physician is most often asked, is that of congestion of the neck, with enlargement and tenderness, and a granular abraded mucous membrane. This inflammation not only appears upon the neck, but almost invariably extends more or less within the cervical canal, and even may involve the whole lining of the uterus, though this is believed to be quite rare.

This granular condition has been cal[illegible] ulceration of the neck, or os uteri," but granulation is more descrip[illegible]ve of the appearance and more truthful of the pathological change. This epithelial disease is the most common form, and is met in practice more frequently than all others combined. As the disease progresses the papillæ increase in size and length, and the diseased patches appear raised above the surrounding healthy tissues. These diseased surfaces are seen covered in their first and milder stages with yellow mucus, which as inflammation progresses in severity, becomes mixed with pus, or is mainly purulent in character; these surfaces also bleed upon the slightest provocation. Yellow leucorrhœa is indicative of this condition, and is probably never present without having some such origin; though it is said that such granular surfaces are sometimes dry, and furnish no such product.

With the *sound* we may learn the size and length of the cervical canal and uterine cavity, and the position of the uterus; may determine if the organ is flexed or retroverted. We may also judge of intra-uterine growths or extra uterine attachments or connections with pelvic tumors. With this condition of congestion or inflammation will also be found *versions* and *flexions*, which are believed to depend upon it as a cause, though versions may accrue from other causes, and not necessarily produce great harm; it is not very uncommon to meet with mal-positions of the uterus which are yet productive of little inconvenience; are corrected occasionally by pregnancy and return again after delivery. The almost universal effect of congestion and inflammation is to increase the weight of the womb, and thereby depress below its natural level. Other causes besides the increase in weight may tend to this result.

*What are our means of cure?*—When there is evidence of inflammation of the mucous membrane only, a judicious use of caustics and astringents will be the most effective treatment which can be adopted. If the inflammation extends to the deeper structures, and is more active in character other means may be premised, since the caustic and astringent applications are found useful mainly when the disease is primarily of the mucous membrane, or has extended from it to the deeper tissues, if they are involved. In the early and more active stage, *baths* and mild saline *cathartics* are often useful in subduing the inflammation and relieving pain preparatory for local applications. The sitz bath, composed of tepid water, and used twice daily, is often servicable, and should be at least mentioned as a means of allaying the earlier urgent symptoms. The temperature of the water may be gradually lowered from tepid to cold, as is found most agreeable and useful.

The caustics and astringents [illegible]st in use are nitrate of silver, persulphate and perchloride of iron, acid nitrate of mercury, chloric and nitric acid, iodine, caustic potassa, and actual cautery. Of these, the nitrate of silver is the almost universal remedy, and though it sometimes does harm, and cannot be used, and sometimes fails altogether, still it will so generally answer the purpose that it may well be regarded as the standing remedy to be discarded only when it fails, or produces injurious effects. The great majority of cases are successfully treated by the judicious application of this remedy alone. It should be applied in solid stick, to the granular surface, which should previously be wiped free from secretions. This application should be made as frequently as the activity of the disease renders necessary, As an ordinary rule nitrate of silver may be applied once a week; to this rule however there are frequent exceptions. The application should be made to the whole diseased surface, extending up the cervical canal and upon the mucous membrane of the cervix, and should be continued until the disease has entirely disappeared; three months is not a long time for the treatment of granulations or ulcerations upon the uterine neck. Nitrate of silver often seems to produce bad effects when first applied; there is more pain, greater amount of discharge, and in severe cases blood is mixed with the mucus, or the blood may be so much in quantity as to mask the mucus, and the patient appear to have hemorrhage; sometimes patients think they are menstruating. A discharge then of bloody serum or blood mixed with secretions is common from the effects of nitrate of silver. This will soon subside, and should not be regarded as unfavorable. Pains also in the back and disturbances of the stomach are not unusual or unfavorable symptoms; they subside without producing serious harm.

When from extension of disease to the deeper tissues, or from other causes nitrate of silver is not successful, and more powerfully stimulant or caustic impression is desired, we may hasten the cure and produce favorable change by more active agents. Chromic acid is a favorite remedy, may be safely and usefully applied in the severe cases of irritation, where the surface is spongy, irritable and vascular, Nitric acid, caustic potassa, and actual cautery are sometimes useful when the milder and less formidable applications fail. The actual cautery since the invention of anæsthesia has lost many of its horrors, and is of great service in some instances. It can be used without great risk and controlled in its action, and on this acounts has advantages over the strong acids and potassa.

In cases of *flexion* and *version*, what means have we of rectifying the condition? We can introduce the uterine sound, and if there is not dilatation of the uterine cavity, so that the sound turns in it, we may replace the womb in natural position, and require our patient to retain the recumbent posture, but this may be repeated as long as we please, and will never permanently rectify the flexion. That practice, of which we sometimes hear, where physicians armed with the uterine probe, replace from time to time what they denominate a "retroverted" or "retroflexed uterus,"

"putting the uterus back in its place," is of no benefit to the patient, and a disgrace to the physician. Flexion of the uterine neck may sometimes be prevented by inducing congestion and swelling, but version will still continue, and the condition is made worse rather than better.

Shall we attempt any form of pessary? There are sterm pessaries which, if carefully introduced, will retain the organ in upright position, but they produce great discomfort, increase the irritation, and sometimes induce acute inflammation. They are occasionally tried by practitioners, but are usually soon abandoned as more troublesome than the disease. Then there are a great variety of other instruments all having the view to elevate the uterus from its depressed situation, and of forming a support upon which it may rest. If the womb is congested and rests upon the perineum in greater degree than natural, it will be difficult to show why it may not as comfortably be sustained there, as upon a ring, air bag, or other substance foreign to the parts, which is itself resting upon the same base, thus constituting at best a mere wedge. All pessaries are liable to produce more or less irritation of the mucus membrane, and increase of uterine disease, and it requires care to use them so as not to do great harm. Occasional cases of great prolapse may be supported in this way, but flexion and version are rarely if ever corrected by this means. Flexion or version connected with irritation, congestion, or ulceration, may reasonably be regarded as depending upon this disease, and the plain object in treatment will be to remove the condition upon which it depends.

Possibly these instruments may occasionally have proved useful, but there is another explanation of their *modus operandi* than the one usually given by the inventor, and acted upon by the prescriber. Their introduction may have a stimulating effect upon the old and indolent action, substituting a new and temporary inflammation, which may be recovered from, for an old and morbid one, which might last for years.

The importance of flexions and versions of the womb has been greatly exaggerated. It is much more remarkable that it should submit to being daily replaced, tipped, twisted and turned by means of the uterine sound, than that it should remain flexed or retroverted with comparative impunity. Believing that the displacement theory is a great fallacy, and being disgusted with the speculation in popular favor and prejudice, which has been successfully prosecuted by appeals which affect so forcibly the popular mind, protest and denial is a duty and a privilege. Dislocation of the womb, by those who know no better, may be regarded as sufficient to account for untold pains and aches, and has been made a pretext for any amount of unnecessary and injurious treatment.

Not to be misunderstood, it may be well to say, that there is no doubt of the existence of the various flexions and versions; that their causes are obvious, and generally acknowledged. It may also be well to state that sudden flexion or version has been induced by falls or unusual exertion and replaced with permanent restoration by position, aided by the hand introduced into the vagina. The importance and necessity of replacing a retroverted uterus, if it occur in the third or fourth month of pregnancy is well understood. These and similar exceptional cases, are not at all considered in the statements concerning flexion and version, and our means of rectifying such positions.

Successful management of uterine disease, depends upon a correct and common-sense regulation of food, air, clothing, exercise, and repose, as well as the proper application of local or specific remedies. The experienced practitioner is fully aware of the importance and necessity of attention to the details of treatment, and will not neglect attention to the general system, and strict regulation of the habits of life.

If ulcerative disease has lasted for years under varying circumstances, perhaps wholly undetected, and improperly treated, it will be presumption to promise a speedy cure, but even under the most adverse conditions we should indulge largely in hope; there are few cases in which we may not entertain high expectations of cure, if we can inspire confidence and courage. Perseverance in treatment will not be continued, unless we can give assurance of ultimate success; and treatment should be commenced with the understanding that we are confident of recovery, though it may be long delayed.

